# Selection of Bifurcated Grafts’ Dimensions during Aorto-Iliac Vascular Reconstruction Based on Their Hemodynamic Performance

**DOI:** 10.3390/bioengineering10070776

**Published:** 2023-06-28

**Authors:** Konstantinos Tzirakis, Yiannis Kamarianakis, Nikolaos Kontopodis, Christos V. Ioannou

**Affiliations:** 1 Department of Mechanical Engineering, Hellenic Mediterranean University, 71410 Heraklion, Crete, Greece; 2 Data Science Group, Institute of Applied and Computational Mathematics, Foundation for Research and Technology-Hellas, 70013 Heraklion, Crete, Greece; 3 Vascular Surgery Department, Medical School, University of Crete, 71003 Heraklion, Crete, Greece

**Keywords:** non-Newtonian blood models, finite volume method, idealized bifurcation, statistical regression tools

## Abstract

During the vascular surgical reconstruction of aorto-iliac occlusive/aneurysmal disease, bifurcated grafts are used where vascular surgeons intra-operatively select the size and the relative lengths of the parent and daughter portions of the graft. Currently, clinical practice regarding the selection of the most favorable geometric configuration of the graft is an understudied research subject: decisions are solely based on the clinical experience of the operating surgeon. This manuscript aims to evaluate the hemodynamic performance of various diameters, D, of bifurcated aortic grafts and relate those with proximal/distal part length ratios (the angle φ between the limbs is used as a surrogate marker of the main body-to-limb length ratio) in order to provide insights regarding the effects of different geometries on the hemodynamic environment. To this end, a computationally intensive set of simulations is conducted, and the resulting data are analyzed with modern statistical regression tools. A negative curvilinear relationship of TAWSS with both φ and D is recorded. It is shown that the angle between limbs is a more important predictor for the variability of TAWSS, while the graft’s diameter is an important determinant for the variability of OSI. Large percentages of the total graft area with TAWSS < 0.4 Pa, which correspond to thrombogenic stimulating environments, are only observed for large values of φ and D > 20 mm. This variable ranges from 10% (for the smallest values of φ and D) to 55% (for the largest φ and D values). Our findings suggest that grafts with the smallest possible angle between the limbs (i.e., smallest parent-to-daughter length ratio) present the most favorable hemodynamic performance, yielding the smallest percentage of total graft area under thrombogenic simulating environments. Similarly, grafts with the smallest acceptable diameter should be preferred for the same reason. Especially, grafts with diameters greater than 20 mm should be avoided, given the abrupt increase in estimated thrombogenic areas.

## 1. Introduction

**Figure 1:**
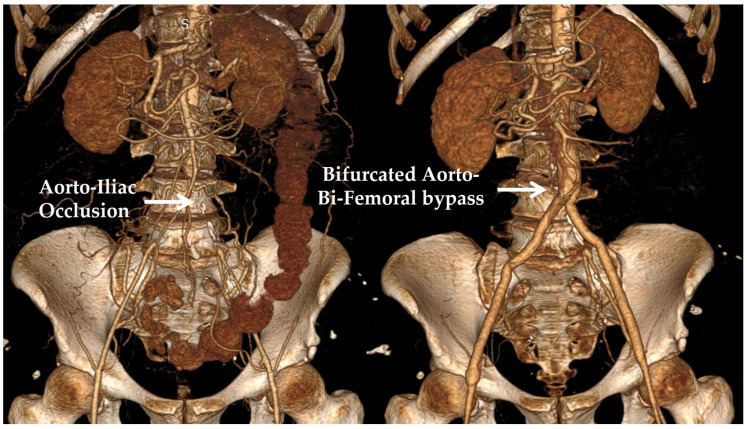
(**Left panel**): A 3D reconstruction of the pre-operative CT angiography of a patient with aorto-iliac obstruction. (**Right panel**): The post-operative CT angiography is shown, displaying an aorto-bi-femoral bypass used for treatment.

Traditionally, vascular surgery has mainly employed surgical procedures where a conduit is used in order to either bypass an occluded arterial segment (atherosclerotic occlusive disease) or replace a diseased and degenerated part of an artery (aneurysmal disease). In one of the most common clinical scenarios, the occlusive or aneurysmal disease affects the aorto-iliac part of the vasculature; in this case, an aorto-bi-iliac or aorto-bi-femoral bifurcated graft is usually employed. Abdominal aortic aneurysms are found in 2–3% of the population older than 65 years of age in the developed world, which, should they escape diagnosis and then enlarge and eventually rupture, can cause an immediate threat to the patient’s life. Aorto-iliac obstructive disease is a distinct pathology that can significantly limit the walking ability of patients, affecting their quality of life and, in extreme cases, posing a threat to limb viability [1]. Considering that the vertical distance between the infra-renal aorta used as the inflow site and the iliac or femoral arteries (target vessels) is patient-specific, a number of options exist regarding the relative length of the main body and the limbs of the graft. Bifurcated aortic grafts are usually manufactured as 20 cm long main tubes that split into two 30 cm long distal tubes; hence, surgeons may intra-operatively shape the graft according to the specific anatomic requirements of each patient. Moreover, aortic grafts are available in a variety of diameter combinations, where the parent tube usually has twice the diameter of the daughter tubes. Typical diameters range from 12 mm (proximal part)/6 mm (distal limbs) to 24 mm/12 mm in increments of 2 mm. Currently, there is no guidance regarding the configuration that should be selected when such a bypass is constructed [2,3]. In most cases, a relatively short main body with two relatively long distal limbs, or a relatively long main body with two relatively short distal limbs, is selected for every patient, based on each surgeon’s preference. An example of a bifurcated aorto-be-femoral bypass of a patient with aorto-iliac obstruction is presented in Figure 1.

On the contrary, the specific anatomy of each patient may dictate the need for a relatively narrow or a relatively wide graft to be used, but a variety of options still exist that are compatible with these requirements. For example, for a 20 mm wide infrarenal aorta, a 16 mm, 18 mm, 20 mm, or 22 mm graft would be considered acceptable. The choice among these options is based on each surgeon’s personal experience and preference, in order to perform a technically sound proximal anastomosis between the native vessel and the synthetic graft. A clinical aspect that has been largely unexplored in the literature is the hemodynamic effects of blood on all alternative graft configurations. Many studies have used computational modeling to evaluate the hemodynamic performance of aortic endografts used during the endovascular repair of AAAs. These examine a variety of outcomes, such as displacement forces, wall stresses, the comparative effects of the “ballerina” versus the standard graft configuration, etc. Although research on the effects of different main body-to-limb length ratios in endovascular stent-grafts has been performed, this has not determined a definite conclusion on the configurations that may be beneficial [4,5]. Moreover, analyses that have evaluated the hemodynamic effects of the relative lengths of the proximal part and distal limbs on surgical grafts are very scarce, with only a recent publication suggesting a possible benefit of a longer main body configuration due to a reduced overall hydraulic resistance [6]. Similarly, although the effect of the inlet diameter has been scarcely examined for endovascular stent-grafts, with the beneficial effect of lower profile devices having been suggested [7], we could not retrieve any studies that have examined the effect of different diameter values on the hemodynamic performance of surgical grafts [8]. To the best knowledge of the authors, there have been no investigations that have associated the hemodynamic effect of alternative graft diameters with the relative length of the proximal/distal parts of the graft.

This manuscript aims to examine the hemodynamic effects of the alternative diameter values of a bifurcated aortic graft and relate them with various proximal/distal part length ratios, in order to provide insights regarding the hemodynamic performance of different geometric configurations and, consequently, to guide clinical practice. A high main body-to-limb length ratio is accompanied by an increased angle between the limbs of the graft, which could be hypothesized to result in flow disturbances and in an unfavorable hemodynamic profile. Additionally, the effect of the inlet diameter of the graft is examined to identify hemodynamically advantageous conditions. For this purpose, a computationally intensive set of simulations is conducted, and the numerical outputs are analyzed with modern statistical regression tools. The paper is organized as follows: Section 2 presents the mathematical framework adopted for the numerical simulations of non-Newtonian fluid flow. Furthermore, it depicts the statistical methodology, which underlies the analysis of the data produced by the simulations. The outcomes are displayed in Section 3 and discussed in Section 4; finally, Section 5 contains the conclusions.

## 2. Materials and Methods

### 2.1. Simulation Setup

Blood is considered an incompressible, non-Newtonian fluid and is simulated by solving the three-dimensional Navier-Stokes (expressed in vectorial form) and continuity equations

(1) $$\begin{array}{l} \rho \left[\frac{\partial U}{\partial t}+\left(U\cdot \nabla \right)U\right]=-\nabla P+\nabla \cdot \tau \\ \;\;\;\;\;\;\nabla \cdot U=0 \end{array}$$

respectively, where U, P, ρ represent fluid velocity, pressure, and density, respectively. For the needs of this study, the shear-rate-dependent viscosity of blood, $\mu \left(\dot{\gamma }\right)\;,$ depends on the deviatoric stress, τ, and the shear rate, $\dot{\gamma },$ as follows

(2) $$\tau =2\;\mu \left(\dot{\gamma }\right)\dot{\gamma }.$$

Various non-Newtonian models have been proposed in the literature for mimicking blood behavior, all aiming to capture deviations from the corresponding Newtonian one. These models include the Carreau-Yasuda [9,10,11,12], Herschel-Bulkley [13,14], and Casson [10,15] models, amongst others. An investigation of the literature reveals that different rheological models are interchangeably used in numerical simulations, with uncertain implications for the results obtained. An attempt to group alternative models in homogeneous clusters has been performed [16], suggesting that data generated from models in different clusters are significantly different. In this work, the Carreau-Yasuda (CY) model is adopted, which is formulated as

(3) $$\mu \left(\dot{\gamma }\right)=\mu_{\infty }+\frac{\left(\mu_{0}-\mu_{\infty }\right)}{{\left[1+{\left(\lambda \dot{\gamma }\right)}^{\alpha }\right]}^{\;\left(1-n\right)/\alpha }}$$

with $\mu_{\infty }=0.00345,\;\mu_{0}=0.056,\;\lambda =1.902,\;n=0.22,$ and α=1.25 [11]. Since blood flow is assumed to be incompressible, its density is considered constant and equal to $\rho =1050\;kg/m^{3}$ [17].

**Figure 2:**
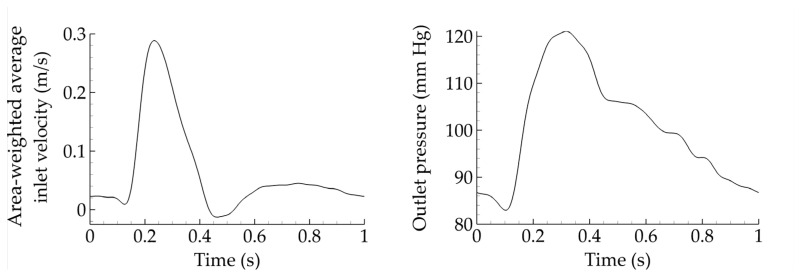
Area-weighted average inlet velocity and outlet pressure for all cases considered.

A commercial finite volume solver is utilized (Fluent 17.2, ANSYS Inc., Canonsburg, PA, USA), with a constant convergence criterion set equal to 10^−5^ and a time step of 0.005 s. Appropriate boundary conditions are considered. Specifically, velocity inlet and pressure outlet waveforms are taken from Olufsen et al. [18] and depicted in Figure 2. Such waveforms constitute a standard set of boundary conditions in blood flow simulations. Finally, the flow split between limbs is assumed to be equal. It should be noted that the assumption of constant inlet velocity and outlet pressure allows us to safely compare all cases and draw meaningful conclusions. However, such constant boundary conditions may not be very realistic, and differences may emerge if one adapts them in terms of the geometrical characteristics of the grafts. In that sense, this assumption should be considered a limitation of the present study.

In all cases, the flow remains laminar and the inlet velocity profile, $U\left(r,t\right),$ is assigned by utilizing a user-defined function (UDF) based on the Womersley method [19], with a corresponding Womersley parameter α (Table 1), given by

(4) $$U\left(r,t\right)\propto \frac{1}{i\;\mu \;\alpha^{2}}{\left(\frac{r}{R}\right)}^{2}\left[1-\frac{J_{0}\left(\alpha \;i^{3/2}\;\frac{r}{R}\right)}{J_{0}\left(\alpha \;i^{3/2}\right)}\right]\;\;e^{i\;\omega \;t}.$$

The velocity profile is thus described in terms of the angular frequency, ω, and Bessel function, J_0_.

**Figure 3:**
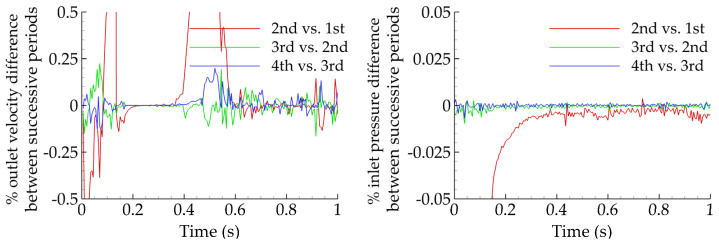
Percentage difference of outlet velocity magnitude (**left**) and inlet pressure (**right**) for various successive periods. The difference does not exceed a few tenths of a percent between periods three and four.

A constant cardiac cycle of one second is assumed for all simulations and four cycles are considered before the results are collected, to ensure that all transient effects are washed out. This can be observed in Figure 3, where the percentage errors for the outlet velocity magnitude and inlet pressure are plotted for successive periods. The error between periods three and four (blue solid) is always less than 0.25%, making period five appropriate for data collection.

**Figure 4:**
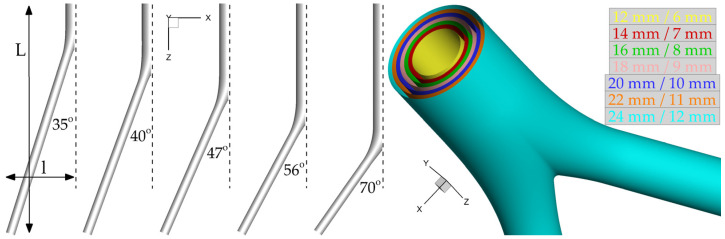
All assumed limb angles and inlet diameters of the idealized geometry. For visualization purposes, only half of the limb angle is shown.

The analyses that follow are based on thirty-five idealized geometries. In all cases, the distance between renal and femoral arteries is fixed (L = 25 cm) and the same holds for the distance between femoral arteries and the midline (l = 7 cm; Figure 4). These values were derived from the evaluation of 10 patient-specific cases treated in our institution. Following the geometric characteristics of commercial grafts, the ratio of the inlet-to-outlet diameter is kept constant and equal to two. Seven diameter configurations D/d (mm) = (12/6, 14/7, 16/8, 18/9, 20/10, 22/11, 24/12) and five limb angles φ (°) = (35, 40, 47, 56, 70) are examined. Assuming a value for the length of the parent vessel y per limb angle, these five cases are characterized by y (cm) = (3, 6, 9, 12, 15), respectively. It is then possible to evaluate the corresponding length of the daughter vessel, x, as using trivial geometric calculations it can be shown that $x\left(cm\right)=\sqrt{y^{2}-50y+674}.$ The five resulting triplets {φ(°)/y(cm)/x(cm)} simulated for each one of the seven diameter configurations are the following: 35/3/23.3, 40/6/20.2, 47/9/17.5, 56/12/14.8, and 70/15/12.2. Assuming rigid walls, all CAD models are built using SolidWorks (Dassault Systèmes, Velizy-Villacoublay, France). Figure 4 presents all examined angles with the corresponding lengths of parent and daughter vessels and all available inlet diameters, which produce the thirty-five simulation cases in total. 

Vascular flows can be analyzed in terms of near-wall hemodynamic parameters over the entire cardiac cycle, T, such as the wall shear stress (WSS) and its most commonly used metrics, the time average wall shear stress (TAWSS, Pa), the oscillatory shear index (OSI), and the relative residence time (RRT, Pa^−1^). Let WSS represent the WSS vector, defined as the dot product of the outward unit normal vector on a surface with the stress tensor. TAWSS is then calculated as the following integral [20]

(5) $$TAWSS=\frac{1}{T}\int_{0}^{T}\left|WSS\right|dt.$$

TAWSS quantifies the tangential force on the vessel wall due to blood flow as the average magnitude of the shear stress, but does not provide any information on the varying frequency of the WSS direction. In order to describe the oscillatory nature of flows, the non-dimensional OSI introduced in [20] is formulated as follows

(6) $$OSI=\frac{1}{2}\left(1-\frac{\left|\int_{0}^{T}WSS\;dt\right|}{\int_{0}^{T}\left|WSS\right|\;dt}\right),$$

with 0≤OSI≤0.5. Flows characterized by no cyclic variation of WSSB, such as uniaxial flows, correspond to OSI=0, while flows with no preferred direction, where the time average of the instantaneous WSS vanishes, yield OSI = 0.5.

Himburg et al. [21] introduced RRT in terms of the two previous hemodynamic markers as

(7) $$RRT=\frac{1}{\left(1-2\cdot OSI\right)\cdot TAWSS}.$$

RRT identifies regions of high-particle residence time close to the wall domain. The three aforementioned metrics have been associated with various diseased states, such as thrombogenic stimulating environments for TAWSS < 0.4 Pa, OSI > 0.3, and RRT > 10 Pa^−1^ [20,22,23,24,25].

### 2.2. Mesh Generation and Convergence

**Figure 5:**
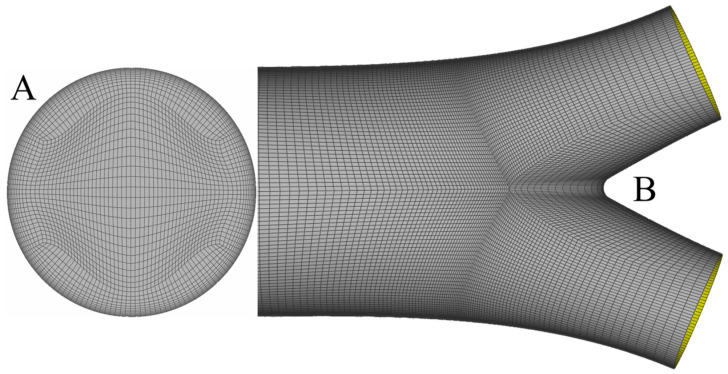
Inlet mesh with the corresponding O-Grid for the construction of the boundary layer (**A**) and the surface mesh close to the bifurcation area (**B**). One observes that mesh density increases while moving towards the bifurcation, to capture non-trivial flow patterns.

In all cases, the solid model is produced by SolidWorks and meshed with ANSA (BETA CAE Systems S.A., Yokohama, Japan) using a pure hexahedral mesh. Hexahedral meshes require a fewer number of elements compared to tetrahedral or prismatic ones for a fixed level of accuracy, as shown in [26]. A sufficient number of elements is clustered close to the wall in order to capture high-velocity gradients. Figure 5 presents the inlet mesh (A) and part of the surface mesh (B) close to the bifurcation area.

To assess mesh convergence, four meshes are constructed with a successive increase of elements by a factor approximately equal to two. Since it is not possible to validate convergence for all thirty-five simulations, one is selected as the benchmark. The chosen case corresponds to the triplet {φ(°)/D(mm)/d(mm)} = {70/12/6}, which is characterized by a significant boundary layer due to the smallest available diameter and non-trivial flow effects as a consequence of the largest available limb angle. An error threshold of 1% in TAWSS, OSI, and RRT is adopted for mesh convergence. The results are summarized in Table 2 and Table 3; one can clearly observe that the required threshold is achieved with the so-called fine mesh.

**Figure 6:**
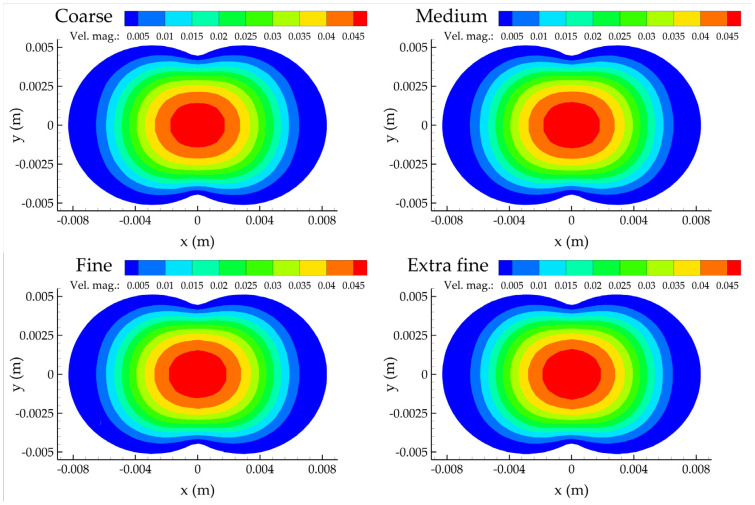
Contour plots of the velocity magnitude at the z = 0 plane for the four mesh refinements.

Figure 6 presents the contours of the velocity magnitude for all meshes considered at the z = 0 plane, defined as the normal to the inlet plane where the three centerlines meet. No apparent differences can be observed; they all share the same qualitative features. The velocity field is characterized by small values close to the wall due to the no-slip boundary condition, and with larger values at the center of the computational domain.

**Figure 7:**
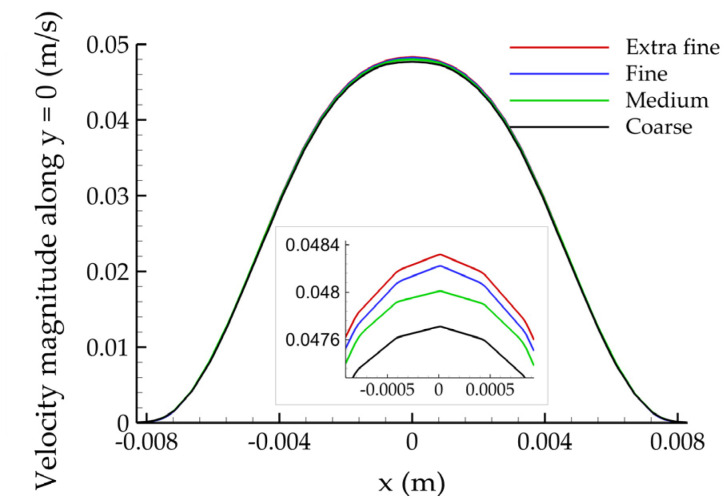
Plot of the velocity magnitude along the y = 0 line of the z = 0 plane for the four mesh refinements.

Figure 7 depicts the velocity magnitude along the y = 0 line of the z = 0 plane. Observing the (magnified) close-ups maxima, it is clear that the solution converges to a constant value with increasing mesh density.

### 2.3. Statistical Analysis

The associations of hemodynamic variables with D and φ are quantified with second order regressions that allow for curvilinear and interaction effects. The general linear (in parameters) specification predicts each response $\hat{y}$ as a curvilinear surface that depends on the levels of D and φ, and uses six unknown parameters, namely $\beta_{0},\beta_{1},\;\ldots ,\;\beta_{5}.$ Specifically, parsimonious variants of the following predictive model

(8) $$\hat{y}=\beta_{0}+\beta_{1}\cdot D+\beta_{2}\cdot \varphi +\beta_{3}\cdot \frac{D^{2}}{1000}+\beta_{4}\cdot \frac{\varphi^{2}}{1000}+\beta_{5}\cdot \frac{\varphi \cdot D}{1000}$$

are evaluated, using each hemodynamic variable (e.g., averaged TAWSS, OSI, and RRT for the whole graft) as the response $\hat{y};$ divisions with 1000 in (8) ease the interpretability of the estimated coefficients without essentially affecting the results. The specification shown above is coupled with backward elimination, a model-building procedure based on the corrected-Akaike information criterion (AICc). AICc leads to improved model-building decisions relative to conventional AIC, especially in small samples [27]. Statistical computations utilize the R packages MuMIn [28] and quantreg [29].

The second-order specification (8) is estimated by the widely adopted least absolute deviations (LAD, or median regression) estimator [29], which can be easily modified to lead to quantile-specific predictive models. The latter are useful tools to assess heteroscedastic associations, with varying levels of uncertainty for different levels of predictors. The reported standard errors for coefficient estimates are computed with a computationally intensive bootstrap procedure, which does not rely on distributional assumptions [30]. Preliminary analyses show that the bootstrap leads to significantly different (typically larger) standard error estimates relative to the frequently applied Gaussian-based ones. This finding suggests that the normality assumption is suboptimal for the analyzed data. Finally, the reported goodness-of-fit, GoF, metric is median-regression appropriate: the variability of residuals relative to $\hat{y}$ is quantified with MAD, the median absolute deviation from the median.

## 3. Results

**Figure 8:**
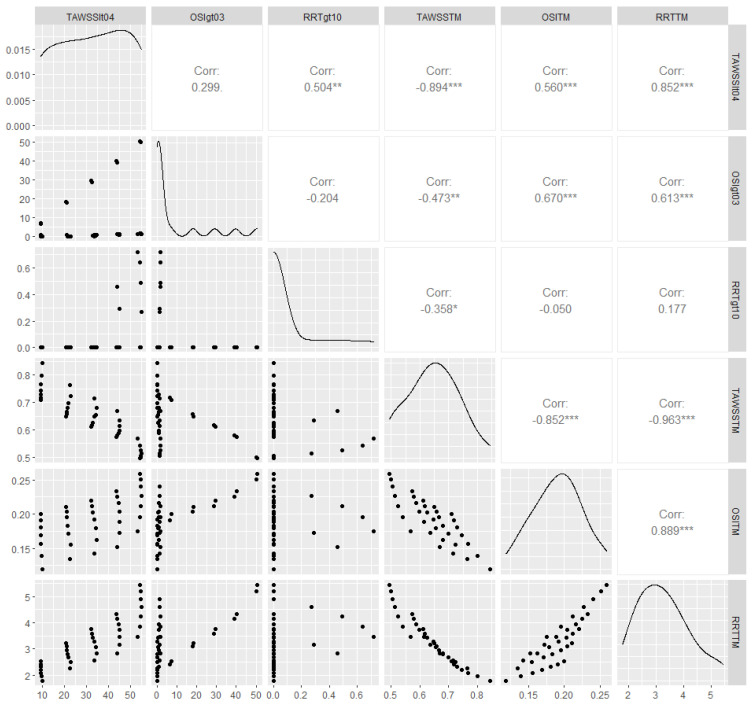
Scatterplots (lower diagonal), density plots (diagonal), and estimated Pearson’s correlation (upper diagonal) for the hemodynamic variables reported in Table 4; weak, moderate and strong evidence against the null hypothesis of zero linear association is depicted with *, ** and ***, respectively. TAWSSlt04, OSIgt03, and RRTgt10 denote, respectively, the percentages of the total graft area with TAWSS < 0.4 Pa, OSI > 0.3, and RRT > 10 Pa^−1^. Similarly, TAWSSTM, OSITM, and RRTTM correspond to TAWSS_TM_, RRT_TM_, and OSI_TM_, respectively.

The analyzed data are summaries of the hemodynamic variables, which correspond to alternative combinations of graft diameters (D/d) with limb angles φ. Table 4 and Figure 8 depict outlier-robust 10% trimmed means (TM) for TAWSS, OSI, and RRT for the thirty-five idealized geometries. Pearson’s rho, a measurement of linear association, is also shown in Figure 8. In accordance with previous studies [13], area-weighted averaged TAWSS (TAWSS_TM_) is strongly, negatively associated with the corresponding values of both averaged RRT (RRT_TM_) and averaged OSI (OSI_TM_). In fact, the correlation of TAWSS_TM_ with RRT_TM_ is almost perfect, which suggests that analyzing one variable from this pair is sufficient. Thus, the following statistical models use TAWSS_TM_ as the response.

**Figure 9:**
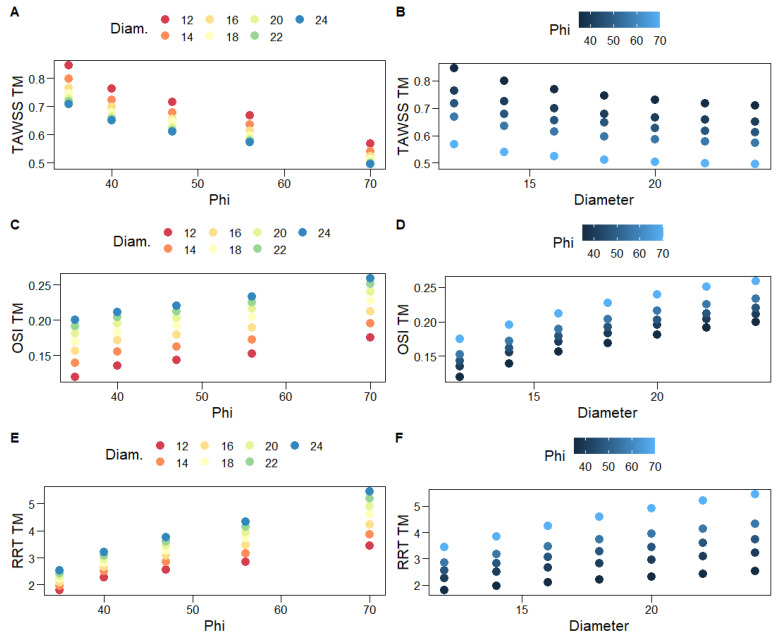
Scatterplots for the associations of hemodynamic variables with graft limb angles, φ (**A**,**C**,**E**), and diameters, D (**B**,**D**,**F**). Different colors are used for increasing levels of D (**A**,**C**,**E**) and φ (**B**,**D**,**F**).

Figure 9 shows scatterplots for the associations of hemodynamic variables with graft limb angles, φ, and diameters, D. One can clearly observe the heteroscedastic, negative curvilinear relationship of TAWSS_TM_ with both φ and D: the variance of the observed TAWSS_TM_ decreases as φ and D increase. As expected, given the abovementioned, strong negative correlations for both OSI_TM_ and RRT_TM_ are positively associated with φ and D. A general second-order specification, such as (8), is deemed adequate to capture the variability of the averaged hemodynamic variables. Indeed, the full second-order model explains the vast majority of the variability of TAWSS_TM_ (Table 5). Limb angles constitute a relatively more important predictor, as a quadratic model solely based on φ explains approximately eight times the variability explained by a quadratic model based on D alone (Table 5). The opposite result is observed for OSI_TM_; namely, graft diameters are more important relative to φ in explaining the variability of OSI_TM_, although the optimal specification is linear; hence, it does not contain the φ^2^ term.

The percentages of the total graft area with TAWSS < 0.4 Pa, OSI > 0.3, and RRT > 10 Pa^−1^, which correspond to thrombogenic stimulating environments, are reported in Table 4 and are depicted in Figure 8. The three percentages are positively correlated, albeit the bivariate association between the TAWSS < 0.4 Pa with the OSI > 0.3 percentages is not strong enough to be of statistical significance. In most simulation scenarios, the observed area percentages with RRT > 10 Pa^−1^ are negligible (Table 4, Figure 8); thus, second-order regression models are not applied in this case. Notably, very large percentages are only observed for large values of φ (56°, 70°) and D < 20 mm; the larger the diameter the worse the result, as the total area of the thrombogenic stimulating environment significantly increases. Similarly, percentages of the total graft area with OSI > 0.3 are negligible for grafts with D < 22 mm; the larger the diameter, the worse the result, with negligible effects of φ.

**Figure 10:**
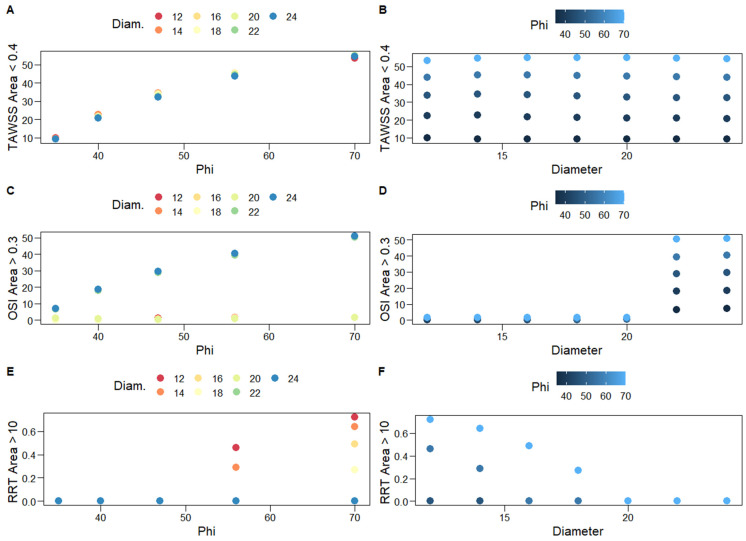
Scatterplots for the associations of the percentages of the total graft area with TAWSS < 0.4 Pa (**A**,**B**), OSI > 0.3 (**C**,**D**), and RRT > 10 Pa^−1^ (**E**,**F**) (thrombogenic stimulating environments) with graft limb angles, φ, and diameters, D. Different colors are used for increasing levels of D (**left column**) and φ (**right column**).

On the other hand, the percentages of the total graft area with TAWSS < 0.4 Pa clearly depend on limb angles (Figure 10A); a quadratic model based on φ alone practically captures all of their variability, whereas the diameter is not a significant predictor (Table 5). Specifically, the simple, φ-based quadratic model is shown below

(9) $$\hat{y}=-1\underset{\left(5.241\right)}{07.803}+\underset{\left(0.205\right)}{4.380}\cdot \varphi -\underset{\left(1.895\right)}{29.442}\cdot \frac{\varphi^{2}}{1000}$$

The model explains approximately 97% of the variability of these percentages (bootstrap standard errors are presented in parentheses, below parameter estimates). This simple specification can be utilized to predict the percentages of the total graft area with TAWSS < 0.4 Pa for unobserved levels of φ, which lie within the examined levels (35–70°).

**Figure 11:**
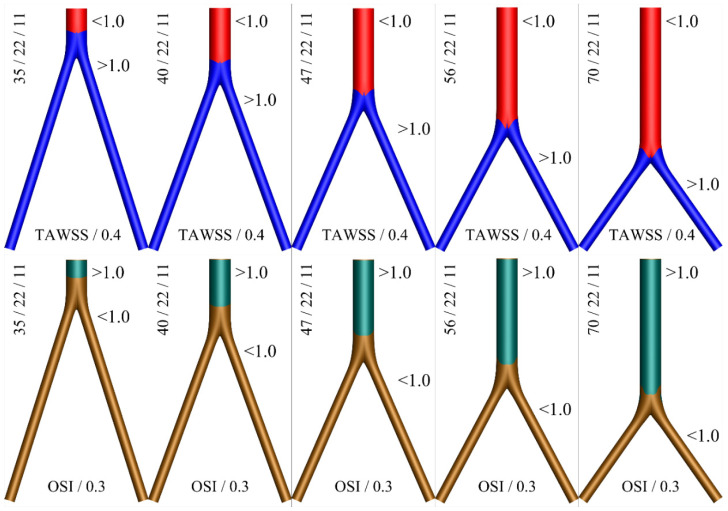
Two color contours for thrombogenic conditions as expressed by the variables TAWSS/0.4 < 1 (**top**) and OSI/0.3 > 1 (**bottom**), for D = 2d = 22 mm. The (blue) red and (brown) green colors depict the (non) thrombus prone areas.

A color map displaying thrombogenic regions along the surface of the idealized geometries for various angles is shown in Figure 11. Based on this graphical representation, the hemodynamic advantage of the short main body (small angle φ) configuration can be observed.

## 4. Discussion

This work is among the few that have utilized advanced, outlier-robust statistical models to analyze blood flow simulations [13,31]. The results presented in the previous section demonstrate that associations between response variables that summarize hemodynamics with a graft’s main characteristics, namely its limb angle and diameter, can be captured with second-order predictive specifications, which include interaction effects. On the other hand, simple linear models are inadequate in general for that purpose. Furthermore, Section 3 carries practical implications when alternative graft choices are available for a patient. Specifically, when accepted grafts only differ regarding their limb angles, it is clear that the one with the smallest φ should be selected; for all examined cases, our analyses revealed that the larger the angle, the larger the percentage of the total graft area that corresponds to the thrombogenic simulating environment. Similarly, when alternative accepted grafts only differ regarding their diameters, the one with the smallest diameter should be selected, especially if the set of accepted grafts includes cases with D > 20 mm, given the abrupt increase in thrombogenic areas observed in Table 4. Actually, Table 4 and Figure 10 strongly suggest against accepting grafts with D > 20 mm when the set of accepted grafts includes cases with smaller diameters. Obviously, when alternative accepted grafts differ regarding both angles and diameters, practitioners should choose the one that corresponds to minimum φ and D.

The abovementioned findings carry several clinical implications. First, regarding the open surgical reconstruction of aortic occlusive or aneurysmal disease, our results indicate that the proximal part of the bifurcated graft should be cut as short as possible, leaving two long distal limbs. This is the configuration that results in the smallest angle φ, which is hemodynamically advantageous. It should be stressed that these grafts are typically intra-operatively shaped by the operating physician. Thus, rendering a variety of different configurations is feasible, although there is currently no evidence to guide clinical practice and these configurations are interchangeably used [6]. A possible advantage of a longer parent tube outline would be the capability to use an aortic endograft in the case of a proximal or distal pseudoaneurysm in need of a secondary intervention. This is important in an era where endovascular techniques are constantly developing [32,33]. Nevertheless, the design of some of the modern endografts would make it possible for a main body length as short as 15 mm to be treated by endovascular means (i.e., the Altura endograft-Lombard Medical, Didcot, Oxfordshire, United Kingdom) [34,35].

Additionally, the diameter of the surgical graft is chosen based on the native aortic diameter, but again, a variety of choices could be deemed appropriate, without any evidence being available to guide physicians. According to the present results, the lowest diameter graft that would be considered suitable should be chosen. It should be noted that a previous publication from our group indicated that, in terms of the parent-to-daughter limb length ratio, a longer parent tube configuration could be advantageous from a hemodynamic perspective, due to the reduced overall hydraulic resistance, as calculated by Poiseuille’s law [6]. This analysis was hampered by the fact that Poiseuille’s law is an oversimplification of hemodynamic simulations, which does not apply to pulsatile flow conditions and non-Newtonian fluids. Therefore, the current approach is methodologically advantageous and more realistic, giving credibility to the present results.

Our findings may carry implications not only for open surgical repair, but also for the endovascular treatment of aortic pathologies. Currently, a variety of aortic modular endografts are used for the treatment of AAAs. Each system has its own anatomic requirements, but these are mostly similar between different devices. In many cases, a variety of devices can be used to treat a given aortic pathology, which are interchangeably used [35]. The design of these endografts can be grouped into three broad categories. The first and most common is where a short main body around 5 cm long splits to two iliac limbs [36]. The second uses an anatomical fixation of the main body into the native aortic bifurcation, where the main body has the length of the native infra-renal aorta [37]. The third is where there is no main body, just two limbs that are simultaneously deployed from the aortic neck to the common iliac arteries [34]. The findings of this work would favor the last design as being hemodynamically advantageous, while the design of anatomical fixation would be the least desirable from a hemodynamic perspective. Regarding the oversizing of endoluminal grafts into the proximal aortic neck to achieve adequate sealing, a 10–30% rate is typically used. Excessive oversizing has been proposed to result in neck-related complications; this could be hemodynamically unfavorable according to our findings [38].

As with the majority of studies, the design of the current work is subject to limitations. The first is the assumption that the vessel walls are rigid. Even though this may be close to reality, the expected effect of graft compliance is not considered. Secondly, additional geometric features, such as graft bending and twisting, as well as graft limb tortuosity, may also alter the presented results. The fact that simple idealized geometries have been taken into account limits the applicability of the present results, since other morphometric indices that might have a significant effect on the simulation results were discarded. Nevertheless, such a methodology is able to better delineate the effect of the different angles and diameters on hemodynamic simulations, which would not be possible in the presence of a plethora of confounding geometric variables encountered in patient-specific anatomies. Moreover, these are the only factors that the operating surgeon can modify, and therefore, the current methodology allowed a relevant and practical conclusion to be produced. Additionally, the flow split between the limbs is not expected to be exactly equal, but nevertheless, it still remains a reasonable assumption.

Finally, the set of thresholds that have been chosen to define a hemodynamically thrombogenic environment may be suboptimal due to the lack of an adequate definition in the existing literature. Indeed, the available data mostly refer to atherogenic hemodynamic conditions, which cause endothelial damage and favor the formation of atheromatous plaque. In the present study, this would not be relevant since the hemodynamic environment inside a graft is our main focus. Nevertheless, recent reports that have studied the potential for thrombus formation inside the branches of custom-made endografts used during EVAR suggest values close to those used in the present study [25,39]. Despite the variability of thresholds reported in previous studies, the values that have been selected here closely approximate those most commonly used in the literature. Future studies along the research path included here include a statistical analysis of the effects of the dimensions, morphology, and angle bifurcation on the hemodynamic change of different geometries, such as patient-specific aneurysms [40,41].

## 5. Conclusions

The current study examines the hemodynamic behavior of various bifurcated aortic grafts for varying levels of proximal/distal length ratios and limb angles. Thirty-five cases are simulated with computational fluid dynamics techniques, assuming a non-Newtonian blood behavior, described by the Carreau-Yasuda rheological model. Our results indicate that grafts characterized by the smallest possible diameter and limb angle present a hemodynamic advantage and should be preferred by practitioners and clinicians.

## Figures and Tables

**Table 1:** Mean and peak Reynolds number as well as Womersley parameter for varying levels of the inlet diameter.

Inlet Diameter, D (mm)	Mean Re, Re_mean_	Max Re, Re_max_	Womersley Number, α
12	237	1055	8.29
14	277	1230	9.68
16	316	1406	11.06
18	356	1582	12.44
20	395	1758	13.82
22	435	1934	15.21
24	474	2110	16.58

**Table 2:** Details of the four meshes examined for the triplet {φ(°)/D(mm)/d(mm)} = {70/12/6}. The table presents the total number of elements, as well as the number of boundary layer (BL) levels and the minimum element size on BL.

Mesh	# of Elements	BL Levels	BL min (mm)
Coarse	151,200	5	0.1504
Medium	252,720	9	0.0538
Fine	512,730	14	0.0203
Extra fine	1,041,692	21	0.0051

**Table 3:** Area-weighted average results and percentage errors for TAWSS, OSI, and RRT. All errors are calculated with respect to the extra fine mesh.

Mesh	TAWSS Result (Pa) Error (%)		OSI Result Error (%)		RRT Result (Pa^−1^) Error (%)	
Coarse	0.604379	5.79%	0.152441	7.36%	3.350175	−3.40%
Medium	0.630215	1.76%	0.160095	2.71%	3.273128	−1.02%
Fine	0.639206	0.36%	0.163306	0.76%	3.245296	−0.16%
Extra fine	0.641492	-	0.164552	-	3.240018	-

**Table 4:** Summary statistics of hemodynamic variables for different combinations of graft diameters (D/d) with limb angles φ; 10% trimmed means of calculated TAWSS (TAWSS TM), OSI (OSI TM), and RRT (RRT TM) are reported, to eliminate the effect of outliers.

φ	D/d	TAWSS TM	% Area with TAWSS < 0.4 Pa	OSI TM	% Area with OSI > 0.3	RRT TM	% Area with RRT > 10 Pa^−1^
35°	12/6 mm	0.845	9.87	0.119	0.00	1.792	0.00
	14/7 mm	0.799	9.42	0.139	0.00	1.955	0.00
	16/8 mm	0.767	9.22	0.156	0.00	2.087	0.00
	18/9 mm	0.745	9.18	0.169	0.00	2.204	0.00
	20/10 mm	0.729	9.15	0.181	1.07	2.315	0.00
	22/11 mm	0.718	9.13	0.191	6.64	2.416	0.00
	24/12 mm	0.709	9.12	0.200	7.08	2.522	0.00
40°	12/6 mm	0.764	22.54	0.135	0.31	2.270	0.00
	14/7 mm	0.724	22.74	0.155	0.00	2.500	0.00
	16/8 mm	0.698	21.79	0.171	0.00	2.668	0.00
	18/9 mm	0.679	21.27	0.183	0.00	2.813	0.00
	20/10 mm	0.666	21.02	0.195	0.81	2.959	0.00
	22/11 mm	0.657	20.88	0.204	17.95	3.091	0.00
	24/12 mm	0.650	20.81	0.211	18.53	3.216	0.00
47°	12/6 mm	0.716	33.72	0.143	1.14	2.554	0.00
	14/7 mm	0.679	34.53	0.162	0.91	2.833	0.00
	16/8 mm	0.655	34.18	0.179	0.45	3.073	0.00
	18/9 mm	0.648	33.56	0.192	0.00	3.268	0.00
	20/10 mm	0.626	32.94	0.203	0.54	3.436	0.00
	22/11 mm	0.617	32.54	0.212	28.73	3.594	0.00
	24/12 mm	0.611	32.31	0.220	29.57	3.746	0.00
56°	12/6 mm	0.669	44.04	0.152	1.53	2.844	0.46
	14/7 mm	0.635	45.13	0.172	1.50	3.168	0.29
	16/8 mm	0.613	45.13	0.189	1.34	3.466	0.00
	18/9 mm	0.597	44.93	0.204	1.03	3.728	0.00
	20/10 mm	0.587	44.56	0.216	0.90	3.949	0.00
	22/11 mm	0.579	44.13	0.225	39.25	4.140	0.00
	24/12 mm	0.573	43.75	0.233	40.22	4.322	0.00
70°	12/6 mm	0.567	53.40	0.175	1.55	3.447	0.72
	14/7 mm	0.541	54.51	0.195	1.59	3.850	0.64
	16/8 mm	0.524	54.90	0.212	1.58	4.231	0.49
	18/9 mm	0.512	54.95	0.227	1.50	4.587	0.27
	20/10 mm	0.504	54.85	0.240	1.50	4.910	0.00
	22/11 mm	0.499	54.67	0.251	50.31	5.202	0.00
	24/12 mm	0.495	54.42	0.259	50.77	5.445	0.00

**Table 5:** Coefficient estimates for the second-order regression model presented in (8); missing estimates correspond to terms that are eliminated from an AICc-based backward stepwise algorithm. Bootstrap-based standard errors are shown in parentheses. ΔAICc reports the difference in the levels of AICc, achieved from the final outcome of the model building procedure, relative to the full second-order specification. GoF is the goodness-of-fit metric that evaluates residual variance relative to the variance of the response (1 indicates the perfect fit of the examined model); MAD, an outlier-robust variance estimator is utilized to compute GoF. GoF_φ_ (GοF_D_) is the GoF achieved by a quadratic model based on φ (D) alone.

	TAWSS TM	% Area with TAWSS < 0.4 Pa	OSI TM	% Area with OSI > 0.3
β_0_	1.634 (0.179)	−94.764 (8.472)	−0.072 (0.026)	150.991 (49.801)
β_1_	−0.036 (0.010)	-	1.561 (0.218)	−16.064 (4.581)
β_2_	−0.017 (0.005)	1.946 (0.307)	0.117 (0.032)	−1.364 (0.776)
β_3_	0.592 (0.209)	−12.016 (7.247)	−0.281 (0.046)	377.692 (104.973)
β_4_	0.080 (0.040)	−26.717 (2.327)	-	-
β_5_	0.135 (0.087)	6.466 (4.625)	0.022 (0.016)	97.158 (50.264)
ΔAICc	0	2.537	2.708	2.695
GoF	0.982	0.973	0.994	0.778
GoF_φ_	0.811	0.962	0.302	0.043
GOF_D_	0.104	0.021	0.630	0.568

## Data Availability

Statistical investigations are fully reproducible as all produced and analyzed data are available upon request.

## Nomenclature

U	fluid velocity
P	fluid pressure
ρ	fluid density
τ	stress tensor
$\dot{\gamma }$	shear rate
μ	dynamic viscosity
T	period of cardiac cycle
CY	Carreau-Yasuda
ω	angular frequency
$J_{0}$	Bessel function
Re	Reynolds number
α	Womersley number
WSS	Wall Shear Stress
TAWSS	Time Average Wall Shear Stress
OSI	Oscillatory Shear Index
RRT	Relative Residence Time
BL	Boundary Layer
L	distance between renal and femoral arteries
l	distance between femoral arteries and midline
AIC	Akaike Information Criterion
LAD	Least Absolute Deviations
MAD	Median Absolute Deviation
GoF	Goodness of Fit
GoF_φ_	Goodness of Fit based only on φ
GoF_D_	Goodness of Fit based only on D
